# Soiling of Pig Pens: A Review of Eliminative Behaviour

**DOI:** 10.3390/ani10112025

**Published:** 2020-11-03

**Authors:** Eleonora Nannoni, André J.A. Aarnink, Herman M. Vermeer, Inonge Reimert, Michaela Fels, Marc B.M. Bracke

**Affiliations:** 1 Department of Veterinary Medical Sciences, University of Bologna, 40064 Ozzano Emilia, Italy; eleonora.nannoni2@unibo.it; 2 Wageningen Livestock Research, Wageningen University & Research, 6708 WD Wageningen, The Netherlands; andre.aarnink@wur.nl (A.J.A.A.); herman.vermeer@wur.nl (H.M.V.); 3 Adaptation Physiology Group, Department of Animal Sciences, Wageningen University & Research, 6700 AH Wageningen, The Netherlands; inonge.reimert@wur.nl; 4 Institute for Animal Hygiene, Animal Welfare and Farm Animal Behaviour, University of Veterinary Medicine, 30173 Hannover, Germany; michaela.fels@tiho-hannover.de

**Keywords:** animal welfare, pigs, eliminative behaviour, defecation, pen soiling, hygiene

## Abstract

**Simple Summary:**

The soiling of pig pens has important negative consequences in terms of animal welfare, health, workload, and environmental emissions of pig farming. The aim of this review is to present the state-of-the-art regarding pigs’ normal eliminatory behaviour (i.e., defaecation and urination) and pen soiling, aimed here at solving pen-soiling problems in existing systems, and in a following publication aimed at the design of a more sustainable pig-farming system. To this end, we summarize a large body of literature on pen soiling and pigs’ eliminative behaviour in different farming systems. We propose a “disease framework” interpretation of pen soiling, to help identify its causes, underlying mechanisms, and solutions.

**Abstract:**

This is a comprehensive review on the pigs’ normal eliminatory behaviour (i.e., defaecation and urination) and pen soiling. This review is aimed primarily at solving issues with pen soiling in current systems, and ultimately at the future design of a well-functioning pig toilet, which we intend to elaborate on in a subsequent publication. In this paper, first, normal elimination is described in relation to what is known about its phylogeny, ontogeny, causation, and function, i.e., according to Tinbergen’s four why questions concerning animal behaviour. Then, pen soiling is described as if it were a medical disorder, highlighting its importance, aetiology, symptoms, diagnosis, pathogenesis, treatment, and prevention. Due to its negative consequences in terms of animal welfare, health, workload, and environmental emissions, possible methods to address pen soiling in current systems are described. Probably, pigs do not choose a specific place to eliminate but rather choose the most comfortable place for resting, and avoid eliminating there. We identified four main strategies to reduce pen soiling: (1) reducing the suitability of the designated elimination area to be used for other functions, especially resting or thermoregulation; (2) improving the suitability of other functional areas in the pen to be used for their specific function, such as resting and activity; (3) reducing the suitability of other functional areas to be used for elimination; and (4) improving the suitability of the elimination area for elimination. These prevention strategies and the encompassing disease framework provide a structured approach to deal with pen soiling in existing systems and to support the future design, development, and implementation of a well-functioning pig toilet that can help to achieve some of the main goals of modern pig production, namely reducing environmental emissions as well as substantially improving pig welfare.

## 1. Introduction

Prior to the development of intensive pig farming, which started around 1950–1960 [1], pigs were generally kept in relatively small pens with straw. Nose rings were used to limit rooting activity in small outdoor fields, while outdoor access was partly seasonal, when fields would be turned into mud fields in autumn and winter. In summer, pigs could often dig their own wallow. Pigs could get dirty in these traditional environments, either from lying in faeces or mud [1,2].

In many European countries, where people had been starving at the end of WW-II, the phrase “no more hunger” became a driving force to provide plenty of affordable food for all. This led to the industrialisation and rationalisation of agriculture [3], where farmers were basically told to “forget” about traditional norms and values in farming, and focus instead on net income, which was to be improved by enhancing production efficiency and a larger scale of production. For instance, in 1950, the Netherlands had 271,000 farms. Pigs were kept on 66% of these farms, i.e., almost 179,000 farms had pigs. The total number of pigs was 1.9 million, i.e., 10.6 pigs per farm on average. In 2018, only about 4000 pig farms were left, housing 12.3 million pigs, i.e., about 3100 pigs per farm, and only 7.6% of all farms had pigs [4]. Many European countries underwent a similar evolution of intensification and specialisation.

From the mid-1970s onwards, however, adverse consequences emerged, firstly tied to a period of overproduction and increasing budgetary pressure for the European Union, and later to progressively increasing societal concerns for the environment, animal welfare, and food safety [5]. It was a time when the term “factory farming” reached the wider public following the publication of Ruth Harrison’s book “Animal Machines” [6] and the Brambell Report [7].

Animal welfare concerns often relate to a perceived discrepancy between modern farming and some kind of ideal condition, which is considered to be more animal friendly, such as traditional farming, living in nature, or being kept as a pet [7,8]. For pigs, the discrepancy manifested itself as highly stocked, dark, smelly, and barren pens without straw or outdoor access.

A crucial innovation in housing design was the use of perforated floors. While laying hens were increasingly kept on wire floors in battery cages, slatted floors for pigs were made of metal, plastic, or concrete. Such floors increase animal hygiene, and reduce material costs and labour as regular mucking out was no longer required. Instead, the liquid manure, collected underneath the slats [9], was mechanically pumped out of the slurry pit. The combination of a high number of pigs on a small area, high emissions of ammonia and odorous compounds from the manure pit, and relatively low ventilation rates caused a deterioration of the indoor air quality. In addition, it became virtually impossible to provide rooting substrates, as these would block the liquid manure system [10]. Straw, however, is highly beneficial to pig welfare as it serves various important functions, including bedding comfort for resting (cushioning and thermal comfort), recreational value for rooting and manipulation, and dietary value by providing gut fill [11]. The provision of inadequate enrichment materials has been a persistent welfare problem of intensive pig farming, leading, e.g., to tail biting and routine tail docking [12,13,14]. Further welfare problems related to the use of hard slatted floors in intensive pig farming include claw lesions, bursitis [15,16], incidental casualties when pigs fall into the manure pit and barn fires related to methane production in the manure pit, and the need to reduce ammonia emissions using air scrubbers [17,18].

In organic pig production, where straw is provided, one of the biggest problems is environmental impact [19], in that emissions are more difficult to control due to a larger living area with a larger emitting surface of solid and slatted floors, including the outdoor area [20,21].

Pigs are naturally motivated to separate areas for lying and eliminating [22]. Solutions, such as a pig toilet, which can be defined as a physically separate area within a pig pen, designed for the deposition of urine and faeces, so that, e.g., ammonia emissions and manure smell can be contained, may thus have major benefits for both environmental reasons in pig farming generally, and for pig welfare in intensive pig production in particular. It could do so especially if such a pig toilet would function (more or less) “automatically”, i.e., in accordance with pigs’ behavioural/welfare needs, and with preferably limited labour requirements of the farmer. Such a toilet should effectively prevent soiling of the rest of the pig pen. This would make slatted floors largely redundant except for the toilet area of the pen. An effectively functioning and robust pig toilet would create important opportunities for the design of the rest of the pen, including the use of long straw or similar substrates, e.g., roughage. If so, reduced housing costs (no manure pit needed underneath the barn; reduced need for heating) might become available for improved toilet design and pig welfare, e.g., more straw and (perhaps even outdoor) space. From an operational point of view, in order to allow these benefits, a pig toilet could, for example, be defined as a location in the pen where pigs preferably excrete, which occupies less than 10% of the pen and where more than 95% of excretions take place. This paper addresses normal eliminative behaviour and pen soiling in existing systems, which is mainly a problem in group housing [23]. When pigs are kept individually, they usually are fixed so as to prevent pen soiling. In newer systems, like free farrowing, pen soiling problems mainly seem to arise due to a lack of space [24]. However, since most pigs are kept in groups (either growing pigs or sows housed in groups during gestation), this review will focus mainly on loose housing in groups.

The aim of this review, therefore, is to present the state-of-the-art regarding pigs’ eliminatory behaviour and pen soiling in existing systems, aimed ultimately at the future development and design of a well-functioning pig toilet. To this end, we summarized existing reviews [25,26,27], describing what is known about pig eliminative behaviour in various environments. We first present the pig’s normal behaviour and then describe the issue of pen soiling in existing systems as if it were a medical disorder. We also identify the main gaps in the knowledge, and propose potential ways forward, both in terms of future research and in terms of how to solve current pen-soiling issues.

## 2. Normal Eliminative Behaviour

**Figure 1:**
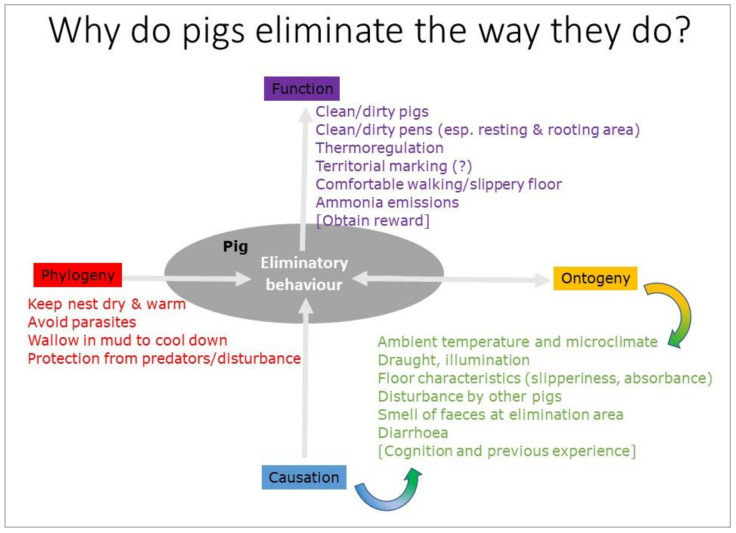
Visual summary of the application of Tinbergen’s framework to explain eliminatory behaviour in pigs in terms of its phylogeny, ontogeny, causation, and function.

This section aims to describe and explain the normal eliminative behaviour of pigs. Elimination, or excretion, is the process of getting rid of material, such as solid waste or urine, from the body. In this paper, the word will be used to refer to defecation and/or urination. Other forms of elimination or excretion, such as vomiting and sweating, fall outside the scope of this review. For the aim of the present paper, the term “eliminative behaviour” will be used to indicate the activities connected with urination and/or defecation. The next section deals with “abnormal” elimination, i.e., pen soiling, also known as pen fouling. In order to describe normal eliminative behaviour in this section, we use Tinbergen’s four why questions to explain behaviour [28], addressing phylogeny (how did the behaviour develop in the pigs’ evolutionary history?), ontogeny (how does the behaviour develop in the animal’s individual life history?), causation or mechanism (what triggers the behaviour; what is the causal chain of events?), and function (what is its adaptive value?; see Figure 1).

### 2.1. Phylogeny

Very little is known about the evolutionary history of eliminative behaviour of pigs. However, the need to use separate areas for different activities is apparently a deep intrinsic behaviour of pigs, which they share with many other animals, in order to avoid contact with their own faeces. Sows have been observed keeping the nest clean, especially during the first few days, by consuming the afterbirths and piglets’ faeces [29]. Piglets start to separate lying and eliminating areas at few days of age, independently of the presence of the mother sow [30]. In observations of domestic pigs in a semi-natural environment, defecation was not performed randomly in space. Pigs tended to eliminate away from both the feeding area and the lying area [31]. The authors identified sites where pigs defecated in the morning after leaving the nest, of which none were closer to the nest than 5 m and none further than 15 m. They also observed that defecations at other times of the day were predominantly left in wide paths, running through the bushes, connecting resting and foraging areas. Incidentally, pigs have been observed defecating during walking, so not even pausing to take posture (Inonge Reimert, personal communication; see below). Perhaps these pigs were in a hurry, e.g., being attacked or in order to keep up with other pigs.

### 2.2. Ontogeny

Young piglets kept on farms already use well-defined areas for elimination, and generally avoid eliminating in the resting area [32]. Newborn piglets begin to consistently eliminate outside the nest at a very young age (≤5 days), and this behaviour appears to be mostly innate, with only a minimum effect played by the sow [30,33]. In organic farrowing pens, piglets eliminate away from the nest and the distance increases as the piglets grow older (Herman Vermeer, personal communication). In farrowing crates, piglets spontaneously, i.e., without any direct intervention from the sow, tend to eliminate in the sow’s dunging place especially if it is well lit, and most eliminations occur after suckling and before playing, and less frequently after sleeping and before suckling [33]. Frequently, several piglets of a litter were observed to urinate at the same time, probably due to the fact that suckling usually finished simultaneously for all piglets by the sow getting up or turning on her belly. These findings agree with Vermeer et al. [34], who postulated that farmed pigs seem to have a rather fixed routine: lying, then eating, drinking, defecating and urinating, exploring, and back to lying, where each activity seems to occur in a “designated” area. Similarly, Hacker et al. [35] stated that pigs often drink, urinate, and defecate in a close sequence.

When an outdoor run is available, pigs will first eat, then drink, and then go outside to urinate and defecate [34]. In runs with wallows, pigs placed the majority (more than 75%) of their dung in the outdoor runs, and about 50% in the wallow, eliminating away both from the roughage and the lying area [36]. Besides dunging away from their nest and food, pigs seem to prefer to eliminate in well-lit [37] (i.e., outdoors), draughty, wet, and safe places, e.g., away from conspecifics [36]. Buchenauer et al. [33] reported that the preference for dunging in well-lit places was also observed for nursing piglets, perhaps related to their tendency to perform elimination in relation to activity in lit areas and rest in cover, i.e., more dark areas. Generally speaking, the places that are not preferred by pigs to lie in or feed seem to remain for excretion. However, it would be very relevant to know whether pigs excrete indifferently at any place that is not a preferred lying or feeding area, or that they may also be intrinsically motivated to eliminate in a specific or designated area, such as a pig toilet.

Wechsler and Bachmann [38] described the typical sequence of eliminative behaviour in a commercial pig pen as follows: after entering the dunging area, the pigs typically sniffed, especially the partitions of the pen. They then adopted the elimination posture defined as turning the hind quarters at least 90° around the sniffed spot or taking a few steps to place the hind quarters within 30 cm from the sniffed spot. This preceded 37% of defecations and 27% of urinations. Following elimination, pigs usually sniffed again, this time focusing their attention closer to the floor. The same authors [38] suggested that besides locating the dunging area, the orientation and sniffing prior to elimination may also be investigation of the barrier preventing the pigs from moving further away from the lying area due to a lack of space (see also [30]). Pigs also turned their hind quarters towards a wall before they eliminated. Sub-adult pigs and sows did so more frequently than piglets, possibly indicating an ontogenetic or cognitive development, e.g., to avoid aggressive or other interactions with conspecifics leading to disturbance during elimination [38].

### 2.3. Causation/Mechanism

Petherick [32] observed that pigs tend to eliminate close to the wall or the pen partitions, and particularly in corners. However, the choice of a specific dunging area has been questioned by other authors. For example, Baxter [39] argued that pigs do not have a specific reason for eliminating in a particular location, but they may develop an individual preference for a dunging place, e.g., if they lose their footing during an elimination, they might move somewhere else the following time. Similarly, Whatson [30] showed that the most pervasive factor appears to be soiled bedding—meaning that piglets simply do not eliminate where they rest, nor do they rest where they eliminate. According to Whatson [30], it is very likely that piglets are motivated to move a certain distance, and possibly around a corner or away from a functional area (such as the nest), before eliminating. In doing so, they might respond to a conspicuous change in environmental features, such as the border of the nest, a different floor type, or the presence vs. absence of bedding material. Pigs may prefer to eliminate close to a wall, or move around a corner, to avoid disturbance by pen-mates when assuming the unstable elimination posture [32]. Baxter [39] postulated that pigs seek isolation during elimination either due to the unstable hunchback/crouched position or seeking to eliminate away from the group, as many other animals do. They then find a relatively safe and isolated area in corners and along walls away from the lying area. The accumulation of faeces along the walls of a pen may thus be the result of seeking isolation or the lack of space to move further away from the lying area, and may thus not indicate an inherent attraction to the wall itself [30], implying that the pigs’ elimination near walls may be a consequence of confinement. Elimination near pen walls, especially when the pen has open partitions, may also be marking of the “home range” and communicating territorial limits to neighbours [35].

Dunging away from the lying area could be mediated by light (see above), or by avoiding the smell of excreta while resting [30]. This would agree with the observation that pigs will rapidly lose interest in enrichment materials that are soiled with dung [40,41]. When pigs are housed indoors, they are confined in a particular, and for them rather uncontrollable, environment, which restricts their ability to thermoregulate, especially when ambient temperatures are high [42]. In addition, the climatic conditions provided to a group of indoor-housed pigs may not meet the needs of all individual animals, e.g., some animals can have higher or lower metabolic rates due to differences in health status or production levels [43,44,45]. Warmer floors (solid) are generally preferred for sleeping [27], but at temperatures above the comfort zone (approximately 20 °C at a Body Weight (BW) of 100 kg and approximately 25 °C at 25 kg BW), pigs prefer lying on the cooler slatted floor, and consequently they will eliminate more on the solid floor [46]. Lying on the slatted floor, and especially lying in the elimination area, may indicate that pigs are experiencing thermal discomfort [42]. Most studies agree that, as ambient temperatures increase, pigs first alter their lying behaviour (by lying laterally and on the slatted instead of the solid floor) and then later change their eliminative behaviour, by soiling the original resting area, and not vice versa. For example, Hillmann et al. [47] observed that pigs only lie in the dunging area at high temperatures when thermal adaptation by lying laterally without body contact did not suffice. Calculations of the occupied area of the slatted floor by Aarnink et al. [46] showed that the switch to elimination on the solid floor occurred before the slatted floor was fully occupied by lying pigs. The reason seems to be that at high ambient temperatures, pigs try to lose as much heat as possible. As the occupation level of the slatted floor increases, the radiative heat loss of the pigs decreases and at some point, this effect compensates the extra heat loss from conduction and convection by lying on the slatted floor. The same study showed that the next step of thermo-regulation is to wallow in urine on the solid floor. Often, it was seen that at very high temperatures, pigs urinated on the solid floor and directly went to lying in their own urine. This was also found by Huynh et al. [48] and it agrees with another study by the same authors [49] suggesting that pigs may be eliminating on the solid floor at high temperatures due to pigs lying on the slatted floor hampering other pigs from reaching the slatted floor or finding an unoccupied quiet place to eliminate. This would indicate a motivation to keep the lying area clean. In line with this, the same study also reported that the temperature at which pigs wallow in urine and faeces was indeed higher than the temperature at which outdoor pigs start to wallow in mud. This indicates a conflict between the motivation to avoid contact with excrements and the need to increase evaporative heat loss by getting wet. As a further confirmation, Damm and Pedersen [50] observed that the highly motivated nest-building behaviour did not alter the ability of preparturient gilts to differentiate functional areas, with significantly more urinations and defecations taking place in the activity area than in the resting area, even in the last 24 h before parturition, when the frequency of elimination was high. Similarly, group-housed sows kept in feeding stalls would hold their faeces for several hours post feeding until being released from the feeding stalls and then able to eliminate further away from the feeding/resting area (Herman Vermeer, personal communication). Thus, pigs may not choose a specific elimination area but seem generally quite highly motivated to keep their lying and feeding areas clean, and they tend to eliminate away from the group. However, while the effects of temperature and space availability have been described, it is less clear to what extent other factors, such as enrichment materials, olfactory cues from other pigs, and walking distance, may affect eliminatory behaviour. Lastly, to our knowledge, only a few studies [38,51] recorded urination and defecation separately, and they reported differences in daily pattern and in behavioural sequence. Ocepeck et al. [51] observed that daily patterns of urination were more prevalent around 15:00 h while defecation was more prevalent around 14:00 h. With respect to behavioural sequence, Wechsler and Bachmann [38] observed that posturing immediately preceded elimination in 37.1% of defecations but only in 27.4% of urinations. These studies raise the question whether it would be more appropriate to consider urination and defecation separately, also given their different physiology and function, in addition to their frequency and pattern of occurrence [25].

### 2.4. Function

A possible evolutionary function of the pig’s eliminative behaviour is the need to avoid evaporative heat loss in young animals, when getting soiled with urine and faeces. Piglets are very susceptible to hypothermia [32]. A possibly more important function is the more generic need for nest hygiene [30]. Keeping the lying area clean would not only enhance thermal comfort but would also improve hygiene by reducing the risk of spreading enteric pathogens (parasites, bacteria, and viruses) [52]. This also applies to the preference to explore, forage, and feed away from dung [40,41]. Finally, eliminative behaviour may also play a role in marking of the home range [35], although the extent to which pigs are territorial is still subject to debate [25].

## 3. Pen Soiling in Current Systems: A Disease Framework

This section deals with pen soiling with a view of helping to solve this problem both in current pig husbandry as well as in future systems incorporating a pig toilet. This section provides a structured description of pen soiling in existing systems as if it were a medical disorder. Such a description seems to work well for describing behavioural problems generally and has been applied to tail biting in pigs and feather pecking in hens [53]. Medical disorders are typically described in terms of their definition, aetiology (main cause), symptoms, (differential) diagnosis, pathogenesis (mechanism), treatment and prevention, and (economic) importance. Table 1 summarizes the disease framework applied to pen soiling in pigs, as described in more detail below.

### 3.1. Definition

Pen soiling may be defined as a condition in which (parts of) the pigs and/or pens get unduly soiled with faeces or urine, usually due to “pigs changing their eliminative behaviour from occurring in the designated dunging area to the lying area” [26]. However, there is no generally agreed standard as to what is undue soiling, and how it is to be measured, e.g., using an environment-based measure (soiling of the pen) or an animal-based measure (number or degree of soiled pigs). For example, a fully-slatted pen may allow a relatively high amount of elimination in the lying area before being “unduly soiled”. By contrast, a pen with a high percentage of solid floor, and especially when containing a pig toilet designed to reduce ammonia emissions, may be considered as being soiled when only few “accidents” have happened. A further, semantic point regarding the definition given above is who determines what is “undue soiling”, as this may vary between people (e.g., farmers, veterinarians, researchers) and locations in the pen (e.g., lying area, feeding area, outdoor run, wallow and dunging area). Notably, it may also depend on the animal’s point of view since, as discussed above, pigs themselves prefer hygiene too, in any case for lying, feeding and exploration.

An example of an environment-based criterion was suggested by Larsen et al. [54], who defined pen soiling as “pens when at least half of the solid floor [which in the study corresponded to the designated lying area] was wet with excreta and/or urine”. The Welfare Quality^®^ protocol for the assessment of pig welfare contains an animal-based measure, “manure on the body”, based on the percentage of the visible side of the body of individual pigs soiled with faeces (up to 20%, 20–50%, or above 50% [55,56]). Maw et al. [57] proposed a 5-point scale (each point representing 20% of the pig’s surface area) and averaging the sores to obtain a pen-level score. The Welfare Quality^®^ protocol also contains an environment-based measure “scouring”, which is defined as the presence of liquid manure in the pen as an indicator of enteric disorders. Other studies suggested a 0-to-3 scale scoring system for the pen, where 0 = no floor soiling and 3 = high floor soiling [58,59]. Due to the variability in assessment methods, a standardization, together with an evaluation of the intra- and inter-observer reliability, has been advocated [60]. A more comprehensive environment-based definition for pen soiling may be “a pen with a sufficiently large area suitable for lying on a clean, dry floor”. The minimum required lying area could be calculated using the formula A = k.W^0.67^, where A is floor surface, W is body weight, and k is a constant. At k = 0.019 pigs can lie sternally without any free space; at k = 0.027, there is some free space for “normal” lying within the comfort zone; k = 0.046 allows lateral lying without body contact or space sharing [61], but this formula is subject to variation depending on the rearing system, e.g., the type of floor and the presence of bedding.

### 3.2. Aetiology

This section describes the (main) causes underlying the disorder. Based on the literature reviewed so far, there is consensus on the main causes of pen soiling in existing systems, which are identified as: inadequate thermoregulation (esp. an overheated lying area or draught), faulty pen design (e.g., too much activity/disturbance or a slippery surface in the dunging area), and flooring issues (e.g., dirty/slippery floors due to spilling of wet feed in trough feeding systems in partly slatted pens and a lack of draining capacity or slope of the floor) [26]. Overall, pigs first choose the best place to lie; and in this respect, thermo-regulation is very important, as is lying comfort. When the preferred resting area of the pigs does not correspond to the lying area assigned by the farmer, pigs may eliminate in the assigned lying area, i.e., on concrete floors, giving rise to soiling issues.

### 3.3. Symptoms

Typical symptoms of pen soiling include pigs and their lying area being (unduly) soiled with faeces and urine. These two symptoms, i.e., pig soiling and pen soiling, are mostly seen at the same time [56] and they can be defined as “signal indicators” related to reduced animal welfare caused by climatic conditions. Soiling with urine may be less easily visible, but to our knowledge, this does not happen as an independent problem. Soiling of the feeding or exploration area may be a localised problem, i.e., restricted to the specific area. Signs indicative of the underlying problems may include e.g., resting in a laterally recumbent position in the dunging area, panting due to heat stress, and restlessness. Draught on the solid floor causes pigs to lie on the slatted floor despite a relatively low ambient temperature. A reduced hygiene status resulting from pen soiling may lead to impaired animal health [62], especially enhanced transmission of gastrointestinal pathogens and parasites, urinary disorders, mastitis, pleuritis, and lameness [60].

### 3.4. Diagnosis and Differential Diagnosis

This section describes when a condition is properly labelled as pen soiling, and how related conditions are to be differentiated from it.

A diagnosis is only possible when an operational definition is used, e.g., when at least 50% of the lying area or the pigs is soiled with faeces. Ideally, pen soiling should be classified against a benchmark population of a similar pen type. Such data, however, are not currently available. It may not always be easy to determine when pen soiling is “unwanted” from the point of view of the pigs. Sometimes, it may be inferred from background knowledge. For example, since pigs generally prefer to rest on a solid floor [27], lying in the slatted dunging area may not be fully in line with what the pigs want. Similarly, when they rest on a soiled solid floor apparently for the purpose of cooling under heat-stress conditions, this may indicate suboptimal welfare as pigs would wallow in mud at a lower ambient temperature, and thus seem to prefer to wallow in mud compared to urine and faeces [48]. Conversely, however, pigs in fully slatted pens that remain relatively clean under heat stress conditions may not have better thermal welfare as compared to soiled pigs in partly slatted pens. In other words, what is undue pen soiling from the point of view of the pigs also depends on the conditions it is compared with, and it is not clear if farmers should prefer clean heat-stressed pigs over soiled less heat-stressed ones either.

Functional areas may also differ in when they should be classified as soiled. Normally, lying areas, feeding areas and enrichment materials should be soiled the least (and preferably not at all), walkways may be soiled at an intermediate level, and specific dunging areas may be soiled the most, while still considered “normal”. It is not clear if and to what extent a dunging area in a pig pen may be soiled before it would be considered “pen soiling”, but for human toilets, it is clear that these may be considered soiled as well, and this may in principle also apply to pig dunging areas, especially when designed as a pig toilet. It should also be considered that investigating pen soiling on fully or partially slatted floors may lead to an underestimation of the level of elimination outside the toilet area. In this kind of rearing system, it may be necessary to directly observe the animals’ eliminatory behaviour, e.g., to see if elimination is occurring in the lying area [63]. In this respect, it is interesting to point out that, to our knowledge, no official definition of “lying area” exists in the literature.

With respect to the differential diagnosis, pen soiling, defined as being unduly covered with faeces and/or urine, should be differentiated from other possible causes of soiling, e.g., pigs or floors being covered by mud when outdoor access or wallows are provided, enrichment substrate (e.g., earth, peat, compost), water (spillage from the drinkers), or feed. Especially when pigs are fed liquid feed or by-products in a trough, this often results in dirtiness around the feeder and dirty pigs. This type of feed-related pen soiling may also facilitate or lead to “real” pen soiling (with faeces) as floors covered with wet feed may reduce the pigs’ ability to differentiate the lying and dunging areas, and may also reduce the pigs’ willingness to differentiate functional areas due to reduced comfort for resting (wet), walking and/or eliminative behaviour (slippery). Furthermore, feeding wet by-products may result in a reduced faeces consistency, increasing the likelihood of pen soiling.

Something similar may happen when pigs suffer from diarrhoea, e.g., due to nutritional imbalances, though, this would be regarded as “true” pen soiling (as it involves undue soiling with faeces). Several endogenous and medical conditions should also be considered in the differential diagnosis of pen soiling. For example, pigs with a dark skin colour may appear soiled. Conversely, dark-coloured pigs may be soiled with faeces without this being as apparent as in pigs having a pink colour, and soiled pigs may hamper the early detection of skin conditions and skin lesions, such as tail or ear biting. Pigs may also look dirty due to a skin disorder, e.g., greasy pig disease.

### 3.5. Pathogenesis

The “pathogenesis” of a disease describes how a disorder may develop over time and how underlying pathophysiological mechanisms may explain its symptoms. Larsen et al. [26] suggested that pigs may change their resting behaviour and thereafter start to eliminate in the area previously used for resting. For example, in partly slatted pens, when the ambient temperature rises, pigs may shift from resting on the solid floor to resting on the slatted floor, both because slatted floors are cooler and because, if the ambient temperature raises further, the pigs may “wallow” there in dung or urine to cool down by evaporative heat loss. As the slatted floor becomes the new lying area, as it is cooler than the concrete floor even without the dung, the pigs may subsequently keep the new lying area clean by dunging in the previous resting area, as observed by Huynh et al. [48]. Alternatively, according to Larsen et al. [54], the pigs may change their resting behaviour following a change in eliminative behaviour. For example, if eliminative behaviour in the dunging area is thwarted because of a disturbance by other pigs or a slippery floor, the pigs may prefer to eliminate in other areas, such as the resting area, and then choose a different place for resting. It is reasonable to expect that both mechanisms (initially choosing a different resting area and initially choosing a different elimination area) may happen, but probably the choice of the lying area is the most important mechanism.

Pen soiling can be affected by a number of factors related to housing, management, and/or the animals themselves. With respect to housing and management, we can identify two main causal factors: (1) inadequate (uncomfortable) resting area and (2) inadequate (unattractive) elimination area. The resting area can be uncomfortable, for example, because of too high or too low ambient temperatures, absence of (sufficient) bedding, draughts, inadequate flooring (e.g., slippery, wet, hard/rough floors, bedding resulting in overheating in combination with warm weather), and disturbance by other pigs [27]. The elimination area can be uncomfortable, e.g., because of disturbance (for example, due to overstocking and the presence of resources, such as drinkers and enrichment materials, resulting in increased activity in the elimination area), the presence/absence of dung (a clean area may be perceived as a potential lying area and a heavily soiled area may be slippery or be avoided due to, e.g., smell). On the other hand, it has been observed that the presence of a small amount of dung in the elimination area may attract subsequent eliminations (Kees Scheepens, personal communication; [64]).

Other factors that may affect pen soiling include the type of floor, feeding system, stocking density, pen or group size, the presence and type of partitions (open/closed), the presence of slippery or unabsorbent flooring (e.g., causing splashes of urine), and pen shape. Temple et al. [52] visited 91 farms over a 2-year period to assess pen soiling in 5 different production systems for growing pigs in France and Spain. The straw-bedded system presented poorer hygiene than the conventional system. The type of floor was a significant causal factor of pig dirtiness in the conventional system and among intensive Iberian pigs, with more soiling on partly-slatted compared to fully-slatted floors. The feeding system, i.e., liquid feeding, was another causal factor of severe pig dirtiness (on more than 50% of the body) in the conventional system, whereas moderate pig dirtiness (on less than 50% of the body) was influenced by the age of the animals with higher levels of moderate soiling as the animals got older and bigger (thus increasing stocking density in terms of kg/m^2^). In addition, it has been hypothesized that an open partition in the slatted area may stimulate elimination due to some sort of marking behaviour [33,35]. With respect to pen shape, Randall et al. [27] argued that a long narrow pen with an elimination area along a long side provides little scope for either demarcation or isolation from disturbance, whereas an elimination area along a narrow side provides some degree of separation in both respects. Additionally, two-level pens may be more soiled than pens without elevated platforms due to their structure, especially when pigs defecate on the elevated platform [59]. Rearing pigs in large groups or in bigger pens (more space per pig) generates a risk for pen soiling as in (very) large groups, pigs may have multiple lying areas and then essentially chose to eliminate in the lying area of other pigs. Pen soiling was a major issue for Dutch pig farmers who started to rear pigs in (very) large groups (Herman Vermeer, personal communication). Conversely, rearing pigs individually in loose housing or in groups containing pigs of very different body sizes, e.g., in free-farrowing systems, may pose specific pen-soiling challenges, such as soiling of the feeding trough or the nesting area [24]. Bøe et al. [24] found that older parity sows were “cleaner” than gilts and mid-parity sows, and Grimberg-Henrici et al. [23] reported that group-housed farrowing sows (kept in groups as of 6 days post partum) were dirtier than single-housed sows. Less is known with respect to animal-related factors, other than age (see above and below). For example, to our knowledge, no study specifically investigated the effect of different genotypes or individual characteristics on pen soiling. Small differences between sexes have been observed. Buchenauer et al. [33] observed that male piglets eliminate near the walls of the pen more often than females, and Rydhmer [65] noted that entire males were dirtier than castrates and female pigs, and that the pen hygiene was lower.

With respect to age, Hacker et al. [35] and Aarnink et al. [46] observed that pens became significantly dirtier as pigs grew older and heavier (see also [15]). This was probably due to the increase in stocking density (expressed as pig weight per square meter) as pigs grew up, and to the lower thermal comfort zone of heavier pigs, as they disperse heat less efficiently. Meyer-Hamme et al. [15] found that moderately-soiled bodies increased from 9.7% at the start to 14.2% at the end of the fattening period (n = 60 farms). They also found that large pens (>30 pigs/pen) showed a higher prevalence of soiled bodies (15.8%) than small pens (<15 pigs/pen; 10.4%), and moderate manure was less often found on pigs fed using a dry feeder compared to liquid feeding. Thus, also the composition, amount and type of feed, and the way the pigs’ digestive system processes it may affect the type of faeces and pen soiling. Scott et al. [66,67] also found that growing-finishing pigs had poorer pen hygiene when fed a liquid diet compared to pigs fed a dry diet, especially when kept on straw (rather than on a fully-slatted floor), and the soiling effect of straw varied with season. Increased soiling in liquid feeding systems could also be due to soiling of the floor with liquid feed, causing the floor to be less suitable for lying.

Lastly, there are some medical conditions that can affect pen soiling. For example, lame pigs may not be willing, or able, to reach the elimination area; lameness or back pain can prevent them from taking the normal posture for urination or defecation. In theory, it is conceivable that disorders affecting the pigs’ sense of smell (e.g., avian influenza, atrophic rhinitis) could hamper pigs from normal eliminative behaviour and enhance pen soiling. An increased urge to defecate or urinate may also result from medical conditions like diarrhoea and urinary/bladder infections, and conversely, pen soiling may enhance the risk of such disorders [60], but to our knowledge, no scientific studies have tried to disentangle these cause–effect relationships between medical disorders and pen soiling.

### 3.6. Treatment and Prevention of Pen Soiling in Existing Systems

This section focusses on possible interventions to address pen soiling in existing systems. For this, we can distinguish between prevention and treatment. In general, however, surprisingly few studies have specifically addressed preventive or therapeutic measures to deal with pen soiling. To our knowledge, no studies have been carried out on therapeutic interventions to curtail a “pen-soiling outbreak”, but farmers may do a number of things like regular cleaning of the floor, adding bedding material and/or feed on the cleaned floor, creating activity, e.g., by hanging a rope where subsequent fouling is to be discouraged, and reducing draught in the lying area, e.g., by increasing the temperature of the incoming air or changing the airflow pattern.

In general, it seems sensible to recommend implementing preventive measures at an early stage. According to Larsen et al. [54], a change in lying behaviour in one single partly-slatted pen in a room, e.g., a reduced number of pigs lying on the solid floor and an increased number of pigs lying on the slatted floor, can be regarded as an early indicator of pen soiling.

Apart from measures addressing pen soiling directly (like using a sloped floor to drain urine, a slatted floor to let manure pass through, or using dry feeders when soiling results from feeding liquid feed), overall, among the most frequently suggested preventive measures are those aimed at improving (micro-)climatic conditions, e.g., monitoring ambient temperatures at the pig level, removing draughts, improving ventilation, checking spillage from the drinkers, etc.

Since pigs probably do not choose a specific place to eliminate, but rather choose the most comfortable place for resting, and hence will eliminate in the less comfortable area(s) of the enclosure [27], pen soiling may be counteracted (1) by reducing the suitability or necessity of the designated elimination area to be used for other functions, especially for resting or thermoregulation, (2) by improving the suitability of other areas in the pen to be used for their specific function such as resting and activity, (3) by reducing the suitability of other functional areas to be used for elimination, and (4) by improving the suitability of the elimination area for elimination.

Since thermal comfort is a well-known factor affecting the pigs’ choice of resting area, it is generally recognised as the single most important factor to control pen soiling [26]. Thermal comfort zones range from 32 °C for very young piglets to around 16–20 °C for pigs of 30–60 kg and 14–20 °C for finishing pigs and adult females [68]. In the so-called thermal comfort zone, no additional energy is needed to maintain the balance between heat production, which depends on metabolism (which in turn is affected by, e.g., feed intake, feed composition, production, activity, and stocking density) and heat loss, which depends on convection, conduction, radiation, and evaporation [69,70]. Convection, conduction and radiation mainly depend on the temperature difference between the skin and the environment, while evaporation mainly depends on the water vapour pressure difference between inhaled and exhaled air and the respiration volume [71], and when the skin is wet also on the water vapour pressure difference between the wet skin and the environment. Since pigs cannot sweat, they, if possible, compensate this by wetting their skin with water or mud. When this is not possible, they depend on panting, seeking shade, drinking more water, lying down laterally on cooler surfaces (e.g., slatted vs. solid floor) without physical contact to other pigs, in order to cool down and/or reduce their feed intake to reduce heat production. When cold, pigs can avoid contact to the (cold) floor (e.g., lie sternally), seek contact with other pigs (huddle), increase muscular activity (e.g., by shivering), and increase feed intake. Thermal comfort can thus be a major determinant of where pigs will rest and eliminate. The main variables affecting thermal comfort include temperature, humidity, and velocity of the air surrounding the pig; temperature and insulation of the floor; and temperature of the surrounding materials (see, e.g., [46,72]).

Several additional recommendations can be given to prevent pen soiling in existing systems related to thermal comfort and pen design.

(1) The first is to reduce the desirability of the (intended) elimination area as a resting area. One example is the use of studs in the elimination area to prevent pigs from resting there. Aarnink et al. [73] reported reduced lying in the elimination area, reduced soiling of the solid floor, and reduced ammonia emissions with metal studs (cylindrical studs, 5 cm high, 2 cm in diameter, spaced at 20 cm) installed in the elimination area. A less clear example may be installing drinkers in the slatted area [39]. Spillage of water results in a wet, cooler floor that, depending on the temperature, may cause pigs to lie away from it, making it more suitable for elimination. However, pigs may also avoid excreting in proximity of the drinkers because of the elevated level of activity in this area.
(2) The second more welfare-friendly approach is to improve the suitability of other areas in the pen to be used for their specific function, such as resting and activity, instead of reducing the (resting) comfort of the elimination area [25,26]. When kept indoors, this could include, for example, providing enrichment materials in the activity area, installing partitions to help the pigs differentiate between different functional areas, and improving the comfort in the lying area. For example, Huynh et al. [42] found that at high ambient temperatures, the use of a floor cooling system embedded in the solid floor resulted in cleaner pens, fewer pigs lying on the slatted floor, and a better feed intake and growth rate. Similarly, water sprinklers resulted in a drop in temperature near the water nipples and less soiling [74]. When bedding or rooting materials are provided, pigs will tend to eliminate away from them. In rearing systems with outdoor access, Vermeer et al. [34] observed that an outdoor rooting area resulted in improved cleanliness of the whole pen (although in some cases the rooting area was also used as a dunging area), leaving the straw-bedded indoor-area clean and dry. Additionally, Olsen et al. [36] found that in pens with an outdoor run, most dunging took place outside, away from the lying and roughage feeding area. Huynh et al. [75] found that providing an outdoor yard (2.5 × 2 m) to pig pens (2.5 × 3 m) containing groups of 5 pigs in a tropical climate reduced the number of eliminations in the resting area, and that adding an indoor wallow had a similar effect (especially when no yard was provided). Improving one type of comfort, e.g., cushioning in the lying area, might, however, also have drawbacks in other respects. For example, Savary et al. [76] observed that at higher temperatures, synthetic plates and straw in the bedding area resulted in more pen soiling because the pigs choose to lie in the elimination area, to cool down. This is also in line with Fraser [77], who demonstrated that pigs only showed a preference for straw bedding over concrete at low temperatures.
(3) The third approach to deal with pen soiling is to reduce the suitability of other functional areas as an area for elimination. Rearing pigs at high stocking densities poses a general obstacle for the separation of functional areas. So, in general, reducing high stocking densities could counteract pen soiling in existing systems. However, in some conditions, e.g., in young pigs, excessive space allowance in the lying area may increase the risk of pen soiling. In such cases, farmers may (temporarily) reduce the size of the resting area or increase the stocking density, even though this may not always be the most welfare-friendly strategy. Randall et al. [27] observed that in conventionally-kept finishing pigs, a stocking density between 120 and 130 kg/m^2^ resulted in a cleaner lying area as compared to both a higher and lower density. While overcrowding may block access to a separate elimination area away from other pigs, too much space in the assigned resting area may give pigs the (false) impression they have moved away far enough from the area used for resting [26]. With abundant space in the resting area, pigs often defecate in unoccupied corners or against walls [78], and once a location has become soiled it may be more likely to be used for elimination in the future [64].
(4) The fourth and final way to steer eliminative behaviour is to make the intended elimination area more attractive as a dunging place, e.g., using olfactory, optical, and/or auditory cues. For example, contact with neighbouring pigs or even an open view to the surroundings may be used [50], as well as the temporal association between elimination and other behaviours, especially feeding and drinking. Hacker et al. [35] found that pens with closed partitions were cleaner than pens with open partitions, regardless of the water position, ambient temperature (up to 30 °C), and stocking density. In this study, closed pen partitions presumably reduced air drafts around the sleeping area and maintained a temperature gradient between the warmer lying area and the cooler (slatted) dunging area. Furthermore, open partitions in the slatted area may stimulate pigs to mark that area of the pen with dung, thus possibly indicating territorial limits to their neighbours, although there is no clear conclusion on whether pigs are territorial or not [25]. Areas close to open partitions could also be uncomfortable as a lying area, due to a disturbance of resting behaviour by the presence or activity of pigs in the neighbouring pens (André Aarnink, personal communication), or because pigs like to use a closed pen wall to lie down [79]. On the other hand, it is also noteworthy that closed partitions may provide a protected place for elimination, which may be preferred by the pigs. Therefore, the use of open or closed partitions may vary depend on the microclimatic conditions in the pen, on pen design and layout, and on the need to delimitate the resting area and/or the elimination area.

With respect to olfactory cues to improve the suitability of the elimination area, pen soiling may be prevented by placing excreta in the intended elimination area before pigs enter the pen [27], which is in line with the observation that young piglets tend to eliminate in the sow’s elimination area [33]. However, to our knowledge, only one study has investigated inducing dunging behaviour in a specific area of the pen using faeces and urine collected from other pens [68], whereas no study investigated the use of other odorous compounds. Yu et al. [64] observed that urine was not effective (probably due to its high volatility), whereas a device containing faeces placed before the piglets’ introduction attracted the eliminatory behaviour of weaned piglets [64]: In the group with the device, defecation occurred in the relatively small designated area (8.3% of the pen) at a ratio of 75–80%, whereas in the control group, this was 45–49%. The effect was increased when faeces was collected 3 days in advance and preserved in air. Perhaps, also, water can act as a visual, tactile, or chronological cue to elicit elimination. Under certain circumstances, e.g., multiple drinkers and thus limited activity in the drinking area, pigs may prefer to urinate in areas around drinkers, which are prone to spillage [39], especially as the two behaviours, drinking and elimination, are often performed in a close sequence [34,35] and as wet areas like wallows seem to elicit elimination [36]. Huynh et al. [75] also found that an indoor water bath (wallow) in a tropical climate reduced elimination in the resting area, especially in pens without an outdoor yard, with pigs often defecating and urinating inside the wallow. Though wallows may thus facilitate eliminative behaviour, especially under warm conditions, other issues, e.g., hygiene, water use, and ammonia emissions, may prevent their application to reduce pen soiling in current commercial systems. In future system designs, however, e.g., a pig-toilet system, wallows may be feasible, perhaps even integrated in the toilet in order to accommodate the pigs’ natural elimination and thermoregulatory behaviour as well to help them deal with more extreme heat-stress conditions, e.g., related to climate change. If the supply of drinking water is insufficient or if pigs are disturbed in the elimination/drinking area, this may also motivate pigs to eliminate away from the drinkers [27,34]. Without disturbance, however, water seems to facilitate elimination, probably also due to a temporal association with feeding and drinking. This may help to prevent pen soiling using pen design or management interventions. Hacker et al. [35], for example, reported that the farther away the drinker is from the lying area, the farther the dung, and the cleaner the pen. Ocepeck et al. [51] observed that the placement of drinkers outdoors compared to indoors resulted in less pen soiling (30% reduction in the likelihood of both urination and defecation on the solid lying area indoors). In addition, and in line with the observation that pigs deposit part of their excretions on paths between the resting and foraging areas [31,80], in organic sows, delaying the admittance to pasture for half an hour may increase the amount of manure on the paved area, and thus reduce the deposition of manure and mineral losses at pasture [81]. It is, however, worth mentioning that delaying the admittance to pasture is not always permitted in organic pig production.

Although poorly addressed in the literature, flooring design in the elimination area may also help to reduce pen soiling. Floors should not be slippery so as to prevent posturing for elimination (Anita Hoofs, personal communication, Herman Vermeer, personal communication). In addition, other flooring qualities may also be relevant, e.g., in separating functional areas or reducing splashing of urine. Pigs may have preferences similar to horses, which have anecdotally been reported to avoid urinating on hard surfaces, preferentially selecting areas with more absorbent surfaces, such as soft soil, grass, or indoor bedded areas [82], possibly to avoid splashing of urine [83]. Piglets have, furthermore, been observed to respond to a conspicuous change in the feature of the environment, such as being inside or out of the nest, to eliminate [30], and, although no studies investigated this aspect, it is likely that also in older pigs a well-differentiated floor type in the dunging area may facilitate eliminative behaviour in the designated area, and thus reduce pen soiling. A similar effect may result from providing a step down into the dunging area. According to Randall et al. [27], this resulted in cleaner lying areas, particularly at the elimination end. Additionally, the use of a “bedding board” could not only hold the bedding in the lying area but also train pigs to step over the bedding board to eliminate in the designated area, as suggested by Fritschen and Muehling [84]. Lastly, pen shape may play a role, with rectangular pens encouraging better dunging patterns than square pens [84], when the excreting area is located at one short side of the pen, creating a bigger distance between lying and excreting areas. It is not exactly known why this is the case. Perhaps, it allows pigs to orientate and find the elimination area more easily, or it may result from improved lying comfort, e.g., alongside the long wall, or a sense of safety, e.g., since deep pens allow for maintaining a larger distance from the walkway. Another possible explanation is that in a rather narrow deep pen, a better separation can be made between the lying and the elimination area, especially when feeders are placed at one of the short sides of the pen. This would help explain also why, in pens with trough feeding, pen soiling is generally a bigger problem, because the troughs are located on the long side of the pen. Recommending a rectangular pen shape, however, should not be taken too far as too narrow pens may again reduce the opportunities for the pigs to get access to the elimination area. Overall, many measures for preventing pen soiling in existing systems would benefit from a certain flexibility in pen design (i.e., changing the position of feeders, drinkers, rooting areas, open/closed pen partitions, etc.) or even the pen’s hardware, barn layout, or business model (e.g., floor type, feeding system, sprinklers, outdoor access, pen shape, stocking densities, etc.). Existing systems often lack the required flexibility to pursue these solutions, and therefore the most feasible measures farmers may take are often limited, and can be summarized as follows:

(1) Be alert for early signs of pen soiling, especially by noting changes in resting behaviour, and take action at an early stage (e.g., by pen cleaning and providing saw dust in the resting area);
(2) Avoid excessive stocking densities, in order to facilitate the distinction between functional areas;
(3) Improve thermal comfort, especially in the designated resting area, e.g., by checking the ventilation system, reducing draughts, and optimising the microclimate (e.g., by floor cooling);
(4) When animals are allocated to a new pen, favour the correct distinction between elimination and resting areas (e.g., by wetting the designated elimination area and providing dry feed or sawdust on the floor of the expected resting area for at least the first few days) [27];
(5) Remove (or limit) olfactory clues as much as possible by thorough pen cleaning before introducing a new group of animals (Herman Vermeer, personal communication). Alternatively, explore the possibilities to direct them towards the correct dunging area by using olfactory cues;
(6) Provide proper enrichment materials facilitating the use of functional areas, e.g., by enhanced synchronisation of behaviour (synchronised activity and rest), by stimulating activity in areas at risk of pen soiling, and perhaps adding lying comfort in the resting area (cushioning from bedding materials). For instance, providing some exploration material (fresh straw, roughage) or nutrition (e.g., grain, corn, lucerne) in the lying area at times appropriate for activity, e.g., during inspection of the pigs, in order to prevent the lying area from being used as an elimination area.

### 3.7. Importance of Pen Soiling

This section discusses why pen soiling in current systems is important. In medical textbooks, this section usually deals with prevalence and economics. Here, we take a somewhat wider perspective and propose to include not only importance for humans but also for the pigs (pig welfare) and the environment (ammonia emissions).

With respect to the prevalence of pen soiling, Jensen et al. [85] estimated growing pigs kept on partially slatted floors soiled the resting area in between 4% and 9% of pens, and 5–10% of pens required manual cleaning. The economic impact of pen soiling has not been estimated directly, but Renaudeau [86] observed that, in a hot tropical climate, pigs housed in a clean environment consumed more feed and grew faster (when pens were cleaned and disinfected before the beginning of the trial, and then washed daily while the manure pit was emptied every week) compared to pigs housed in the dirty environment (in which none of these actions was taken). Huynh et al. [42] observed an increase in feed intake and growth rate together with a reduction in pen soiling when pen floors were cooled. However, these effects were likely related to the improved thermal comfort and not to pen soiling by itself. Interestingly, Van der Meer et al. [62] showed that pigs kept under poor sanitary conditions (including soiled pens) showed reduced growth rates and a compromised health status (higher pleuritis scores at slaughter and an increased innate immune response and serum haptoglobin concentrations). Courboulay [60] indicated that pen soiling, especially in the case of sows, has been associated with urinary disorders, puerperal mastitis, and a 2.8 elevated risk of lameness, and that it may hamper welfare assessment and management (e.g., the detection of skin lesions).

Pen soiling probably also has a considerable and perhaps often underestimated impact on pig welfare. It is, by itself, an indicator of reduced animal welfare as it implies the presence of factors in the environment that override the pigs’ intrinsic motivation or natural tendency to keep the resting area clean and separate from the elimination areas. Such causal factors include overcrowding, heat stress, inadequate flooring, health problems, and disturbance from other pigs. In addition, pen soiling may lead to a further reduction in welfare when air quality, thermal comfort, skin condition, hygiene, and health status are adversely affected. Loose-housed sows, which were temporarily confined to stalls after feeding (in order to collect faecal samples), held up for several hours, probably to avoid eliminating close to the feeding and resting area of the stalls (Herman Vermeer, personal communication). This indicates a certain level of motivation in these sows to avoid pen soiling. Another indication of this motivation derives from the observation that pigs will wallow in faeces or urine in the resting area at a higher ambient temperature than they would use outdoor wallows [48].

EU Directive 2008/120/EC [87] requires that pigs have access to a lying area physically and thermally comfortable as well as adequately drained and clean, which allows all the animals to lie at the same time. Furthermore, Commission recommendation (EU) 2016/336 [88] specifies that farmers should consider cleanliness (together with enrichment provision, thermal comfort and air quality, health status, competition for food and space, and diet) as part of the risk assessment to reduce tail biting and the need for tail docking. Pen hygiene is thus also a legal requirement.

Lastly, reducing pen soiling is important to limit the impact of pig production on the environment as soiled pens may substantially increase ammonia and odour emissions [46]. When pigs are housed indoors, ammonia emissions are affected both by the proportion of slatted floors and by the size of the soiled solid floor surface area [89]. When pigs have outdoor access, e.g., in organic systems, the manure tends to be deposited in the outside yard. This may increase overall ammonia emissions (when urine and faeces are mixed, especially in manure pits under slatted outdoor yards), and leakage of other nutrients (if pigs eliminate on pasture or soil areas without adequate crop/animal rotation). These emissions from organic farming can sometimes even exceed the standards for regular pig farming [80]. Similarly, Eriksen et al. found that fattening pigs on pasture carry a high risk of nutrient loss, and suggested the adoption of preventive strategies (decreasing stocking density and dietary nitrogen intake, opting for a seasonal production as opposed to a year-round one) [90].

Stimulating animals to deposit excretions in a relatively small location, where it leads to or can be collected with a minimum of ammonia emissions and hygiene problems [25], could be a major step towards more sustainable pig production. In a way, such designs are already in use in some farms. For example, in partially-slatted conventional pens, pigs generally prefer to rest on the solid floor and to eliminate on the slatted part of the pen, provided the ambient temperature is kept within the pigs’ comfort zone [46]. Well-designed dunging areas, perhaps even specific “pig toilets”, could further reduce the need for slatted flooring and thereby allow the use of superior enrichment materials like roughage and long-straw bedding. This would be a major step forward for pig welfare but could also bring further environmental benefits (André Aarnink, personal communication), e.g., when urine and faeces can be collected separately this could not only further reduce ammonia emissions but also provide more valuable nutrients to fertilise fields.

## 4. Conclusions

This paper provides a framework in which the eliminative behaviour of pigs was systematically described by asking Tinbergen’s four why-questions about behaviour (phylogeny, ontogeny, causation, and function) and by describing pen soiling in existing systems as if it were a medical disorder in terms of its definition, aetiology, symptoms, diagnosis, pathogenesis, treatment and prevention, and importance. In addition to its importance, as described in the previous section, for current pig production and pig welfare, we want to emphasise here again that a thorough understanding of this behaviour may also be important or even crucial for the development of a pig toilet (which we will explore in more detail in a forthcoming paper). Getting pigs to use such a designated area for urination and defecation is a challenge that opens up possibilities to considerably reduced ammonia emissions from pig buildings as wells as improved pig welfare, including a better air quality, a more comfortable floor to rest on, the provision of exploration materials allowing natural rooting behaviours and intact tails, and perhaps also the provision of more (outdoor) space and a wallow in the future. In this way, the ultimate goal of eliminating pen soiling may be a sustainable pig-toilet system that eliminates (most of) the slatted floor through the facilitation of normal eliminative behaviour that is well within the boundaries of the pig’s cognitive and adaptive capacities. The possibilities to design such a toilet will be the object of another manuscript, which is at present under preparation.

## Figures and Tables

**Table 1:** Application of the disease framework to pen soiling in existing systems: A summary.

**Definition**	Pigs and/or pig pens get unduly soiled with faeces or urine, usually due to a change in lying behaviour. No agreed standard as to what is undue soiling, and how to measure it.
**Aetiology** **(Main Cause)**	Inadequate thermoregulation (overheated/draughty lying area), faulty pen design (disturbance during elimination), flooring issues (dirty/slippery floors).
**Symptoms**	Pig and pen soiling (mostly seen at the same time) can be a “signal indicator” related to reduced animal welfare caused by climatic conditions. Additional symptoms: resting in the dunging area, panting due to heat stress, restlessness, lying on the slatted floor also at low ambient temperature, impaired animal health (increased transmission of gastrointestinal pathogens and parasites).
**Diagnosis and Differential Diagnosis**	An operational definition is needed for a clear diagnosis (e.g., different functional areas and system specifications (e.g., floor type) may determine when a pen is classified as soiled). Differential diagnosis: faecal soiling to be distinguished from mud, enrichment substrate (earth, peat, compost), feed, diarrhoea, dark skin colour, skin disorders (e.g., greasy pig disease).
**Pathogenesis**	Three possible mechanisms: Inadequate (uncomfortable) resting area: Change in resting behaviour (e.g., due to high ambient temperature) followed by elimination in the area previously used for resting. The choice of the lying area seems to be the most important factor. Inadequate (unattractive) elimination area: Change in eliminative behaviour (e.g., due to disturbance or slippery floors) followed by a change in resting behaviour. This may occur more occasionally or by accident. Animal-related factors: e.g., genotype, individual characteristics, sex, age, medical conditions, previous experience, etc.
Treatment and Prevention *	General advice for farmers to deal with pen soiling in existing systems: Be alert for early signs (e.g., changes in resting behaviour), and take action at an early stage (e.g., clean pens and provide bedding in the resting area); Improve thermal comfort, esp. in the designated resting area (check the ventilation system, reduce draughts, and optimise the microclimate); In newly formed pens, favour the correct distinction between elimination and resting areas (floor of the designated elimination area can be wetted, and dry feed or sawdust can be provided on the floor of the expected resting area during the first few days); Remove (or limit) olfactory clues in the expected resting area as much as possible (thorough pen cleaning before introducing a new group of animals), provide smell of faeces in the designated dunging area; Provide proper enrichment materials to facilitate the distinction of functional areas (to stimulate activity in areas at risk of pen soiling) and if possible, add bedding materials in the resting area (to improve comfort).
**Importance**	Reported prevalence: 4–9% of pens. Pen soiling indicates, by itself, reduced animal welfare (i.e., rearing conditions overriding pigs’ motivation/tendency to keep the lying area clean). Poor hygiene may reduce growth rates, compromise health, reduce welfare and air quality. Legislation requires pigs to be kept in a thermally comfortable, adequately drained and clean area which allows all the animals to lie at the same time. Reducing pen soiling and/or a “pig toilet” could reduce the environmental impact of pig farming, and further improve pig welfare (e.g., by providing straw or roughage on a solid floor) in the future.

* For the purpose of this table, only the most immediately feasible measures for farmers were presented. A more detailed description of measures requiring either modifications in pen design, pen hardware, or even in the barn layout or business model, is given in the text.
